# Direct production of molecular oxygen from carbon dioxide and helium ion collisions

**DOI:** 10.1038/s42004-023-01074-2

**Published:** 2023-12-06

**Authors:** Yaya Zhi, Qiang Guo, Jingchen Xie, Jie Hu, Shan Xi Tian

**Affiliations:** 1 Department of Chemical Physics, Collaborative Innovation Center of Chemistry for Energy Materials (iChEM), University of Science and Technology of China, 230026 Hefei, China; 2 Hefei National Research Center for Physical Sciences at the Microscale, University of Science and Technology of China, 230026 Hefei, China; 3 Hefei National Laboratory, University of Science and Technology of China, 230088 Hefei, China

**Keywords:** Physical chemistry, Atmospheric chemistry, Astrochemistry, Chemical physics

## Abstract

The prebiotic mechanism to produce molecular oxygen (O_2_) in carbon dioxide (CO_2_)-rich planetary atmospheres is of great importance in understanding astrochemical reactions and is potentially relevant to the origin of life on Earth. Here, we demonstrate that, aside from the direct productions of O_2_ by photodissociation and dissociative electron attachment, the low-energy ion-molecule reaction between cationic helium in solar winds and molecular CO_2_ is a noticeable mechanism. Branching ratios of the reaction channels are determined, and their absolute cross-sections are estimated accordingly. The present findings represent a further, indispensable step towards fully understanding the origins of atmospheric O_2_.

## Abstract (web summary)

The abiotic production of molecular oxygen in carbon dioxide-rich planetary atmospheres is important in understanding astrochemical reactions and potentially the origin of life on Earth. Here, the authors demonstrate that the low-energy reaction between helium ions, as found in solar winds, and carbon dioxide produces molecular oxygen.

## Introduction

Before the dramatic rise of atmospheric oxygen during the *Great Oxidation Event* about 2.4 billion years ago on Earth^1,2^, few oxygen molecules (O_2_) already existed in the primitive atmosphere which was nearly full of carbon dioxide (CO_2_). Those O_2_ molecules were exclusively produced via abiotic processes. A widely accepted mechanism about the O_2_ origin in CO_2_-rich planetary atmosphere is the cascade process induced with vacuum ultraviolet (VUV) photon

1a $${{{{{{\rm{CO}}}}}}}_{2}+{{{{{\rm{h}}}}}}{{{{{\rm{\nu }}}}}}({{{{{\rm{VUV}}}}}})\to {{{{{\rm{O}}}}}}+{{{{{\rm{CO}}}}}}$$

1b $${{{{{\rm{O}}}}}}+{{{{{\rm{O}}}}}}+{{{{{\rm{M}}}}}}\to {{{{{{\rm{O}}}}}}}_{2}+{{{{{\rm{M}}}}}}$$

where the product O_2_ can be stabilized efficiently by releasing a third body M^3–5^. A recent experimental study indicated that O_2_ was directly produced in near extreme ultraviolet (XUV) photodissociation^6^

2 $${{{{{{\rm{CO}}}}}}}_{2}+{{{{{\rm{h}}}}}}{{{{{\rm{\nu }}}}}}\,({{{{{\rm{XUV}}}}}})\to {{{{{{\rm{O}}}}}}}_{2}+{{{{{\rm{C}}}}}}$$

More recently, we reported another finding that dissociative electron attachment (DEA) also directly led to the O_2_ formation^7^

3 $${{{{{{\rm{CO}}}}}}}_{2}+{{{{{{\rm{e}}}}}}}^{-}\to {{{{{{\rm{O}}}}}}}_{2}+{{{{{{\rm{C}}}}}}}^{-}$$

Since a large quantity of low-energy free electrons were detected in the atmospheres of Venus and Mars, as well as the current Earth^8–11^, Eq. (3) and relevant DEA processes that were unheeded over a long period should be incorporated into the atmospheric reaction networks.

Besides VUV/XUV photons and free electrons, He^2+^ (alpha particle) and He^+^ are the heavy (respective to H^+^) ions in the solar wind^12–15^. Although the He^+^/He^2+^ collisional reactions were confirmed to contribute to the production of O^+^, O_2_^+^, and CO_2_^+^ in the Martian ionosphere^16,17^, there is still no indisputable evidence about the O_2_ formation^18–20^. Here we demonstrate that O_2_ and O_2_^+^ are certainly produced in the following collisional reactions:

4 $${{{{{{\rm{CO}}}}}}}_{2}+{{{{{{\rm{He}}}}}}}^{+}\to {{{{{{\rm{O}}}}}}}_{2}+{{{{{{\rm{C}}}}}}}^{+}+{{{{{\rm{He}}}}}}$$

5 $${{{{{{\rm{CO}}}}}}}_{2}+{{{{{{\rm{He}}}}}}}^{+}\to {{{{{{{\rm{O}}}}}}}_{2}}^{+}+{{{{{\rm{C}}}}}}+{{{{{\rm{He}}}}}}$$

by the measurements of time-of-flight (TOF) mass spectra and ion velocity maps using a crossed-beam apparatus^21–23^.

**Fig. 1:**
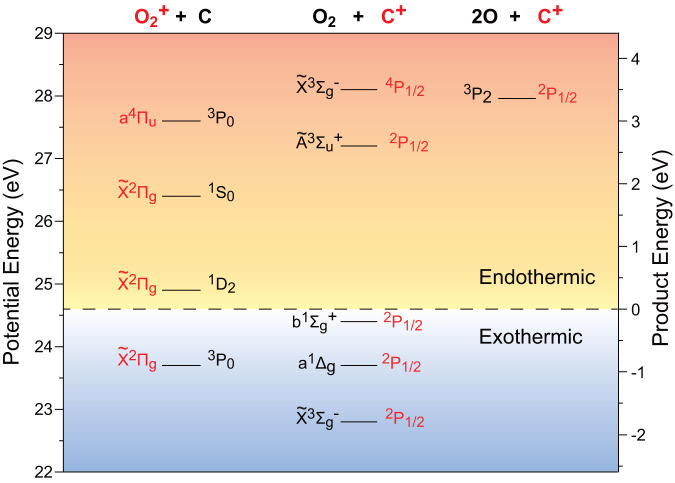
Asymptotic energies of three charge-exchange reactions between He^+^ and CO_2_. The product energy is scaled, concerning the zero value for the resonant charge exchange of the motionless reagents He^+^(^2^S) + CO_2_(X̃^1^Σ_g_). Besides the exothermic pathways, the endothermic processes could proceed if the collision energy is availably transformed.

Previously, the experimental drawbacks such as multiple collisions and ionic yield collection or transportation gave rise to a divergence in whether O_2_ or O_2_^+^ could be produced in the thermal-energy reactions of He^+^ + CO_2_ (refs. ^18–20^), while those technical troubles are settled in the present measurements. On the other hand, the translational energy of He^+^ in the Martian atmosphere spreads over a wide range and depends on its sources, for instance, the velocity of He^+^ is comparable to that of He^2+^ in the solar wind if He^+^ is produced by the charge exchange reaction between He^2+^ and neutral hydrogen^13^. Besides the thermal-energy collisions, He^+^ + CO_2_ reactions at the collision energy of electron volts are particularly interesting, since the products in different electronic states, as shown in Fig. 1, play versatile roles in the subsequent processes.

CO_2_^+^ formed in the resonant charge exchange between CO_2_ and He^+^ can be populated in the ro-vibrationally excited states of electronic state C̃^2^Σ_g_^+^ or the ^2^Π_u_-satellite states, but its spontaneous dissociation is highly preferred due to an electron depletion from the bonding orbital 4σ_g_ or 1π_u_ of CO_2_ (refs. ^24,25^). The channels producing CO^+^ + O and O^+^ + CO were found to be dominant in the thermal collisions of He^+^ + CO_2_ (refs. ^18–20,26^), and as one of their subsequent processes, the photoemission A͂^2^Π → X͂^2^Σ^+^ of the CO^+^ yield may contribute to the comet tail glow^26^. Such scenarios can be further found in Fig. 1. For instance, the phosphorescence from the O_2_ in singlet states (*a*^1^Δ_g_, *b*^1^Σ_g_^+^) was observed in the aurora^27^. The reactions noted in Fig. 1, once authenticated experimentally, should be of fundamental importance toward understanding the oxygen-related processes in planetary atmospheres.

The present experiments were conducted with our home-made crossed-beam apparatus which was recently updated by introducing the three-dimensional velocity map imaging technique of ionic products^22,23^. The TOF mass spectra and velocity distributions of all ionic yields can be obtained simultaneously. The collection efficiency of ionic yields is close to 100%, and each peak in the TOF mass spectrum corresponds to the whole Newton spheres of a certain type of ionic yield. The multiple collisions in the ion flowing or the ion drift of cyclotron resonance^18–20^ are absent or reduced significantly in the crossed-beam arrangement where the pulsed reactant ion beam is perpendicular to a supersonic target beam.

Due to the energy redistribution in a collision, the endothermic channels (their energetic thresholds can be found in Table S1 of Supplementary Note 1) in Fig. 1 are possibly accessed. For example, around 3.5 eV of the collision energy, the reactions leading to O_2_(X͂^3^Σ_g_¯) + C^+^(^4^P_1/2_) and 2O(^3^P_2_) + C^+^(^2^P_1/2_) are allowed in energetics if the collisional or translational energy is completely transformed into the internal energies of the target. However, in the TOF mass spectrometry measurement, the same product, C^+^ as mentioned above, cannot be distinguished from which channel the ion originates. Different ion production channels could be further identified by the ion velocity imaging measurements. In this work, the combinational measurements were accomplished at three collision energies in the reaction center-of-mass (c.m.) coordinate (*E*_c.m._ = 1.94, 2.49, and 4.75 eV).

## Results and discussion

**Fig. 2:**
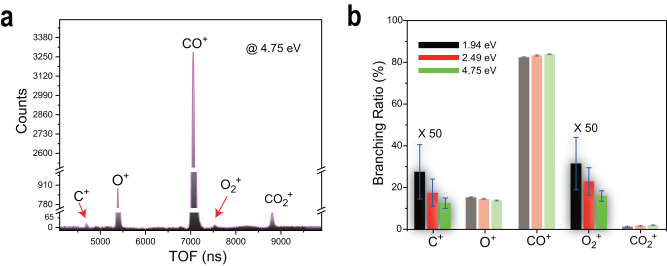
Ionic yields of the collisional reaction between He^+^ and CO_2_ at 4.75 eV (a) and branching ratios (b). **a** Time-of-flight (TOF) mass spectrum is plotted with the real ion counts. **b** Branching ratios (with the statistic uncertainties of the measurements) of O_2_^+^ and C^+^ yields are amplified to be more visible.

The TOF mass spectrum at *E*_c.m._ = 4.75 eV, as a typical example, is shown in Fig. 2a, in which the C^+^ and O_2_^+^ ions are observed clearly but their relative intensities are much lower than those of other ionic yields. Such low signals of C^+^ and O_2_^+^ certainly brought difficulties in the previous measurements^18–20,26^. Here, we further obtained the branching ratios of the reactions and plotted their collision energy dependences in Fig. 2b. A comparison between the present branching ratios and the previous ones^18–20^ is presented in Table S2 of Supplementary Note 2.

In the reactions at the higher collision energies (10–10^3^ eV), the production ratio *γ* (C^+^/CO^+^) was found to gradually increase with the enhancement of He^+^ kinetic energy^24^. Here one can find that the branching ratios of the reactions denoted with Eqs. (4) and (5) decrease with the *E*_c.m._ enhancement. Such different collision-energy dependences should be attributed to reaction dynamics, e.g., diversities of collisional trajectories and dissociation pathways. Usually, the time scale of a charge exchange at the higher collision energy could be shorter than that of molecular vibrational motions. O_2_ can only be formed via the slow atomic roaming^6^ or the bending motion with a large amplitude of CO_2_ (ref. ^7^). Thereby, similar scenarios should exist for the O_2_ or O_2_^+^ product of the He^+^ + CO_2_ reactions at the lower collision energy.

**Fig. 3:**
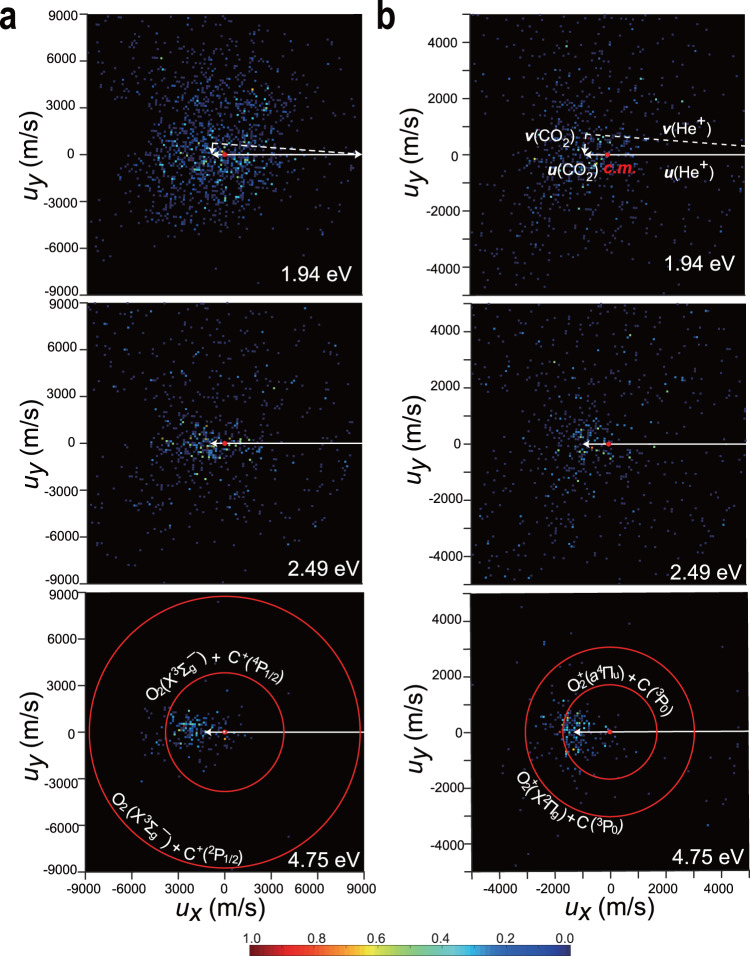
Velocity map images of C^+^ (a) and O_2_^+^ (b). Each velocity image is the projection of three-dimensional Newton spheres of C^+^ or O_2_^+^ to the ion detector. Red circles represent the isotropic distributions for O_2_(X̃^3^Σ_g_¯) + C^+(2^P_1/2_) or O_2_^+(^X̃^2^Π_g_) + C(^3^P_0_) (outer circle) and O_2_(X̃^3^Σ_g_¯) + C^+^(^4^P_1/2_) or O_2_^+^(*a*^4^Π_u_) + C(^3^P_0_) (inner circle) yields that are assumed to be produced in photodissociation of CO_2_^+^ at the center-of-mass (c.m.) of the collision. White lines with arrows show the coordinate transformation from the laboratory (the reactants’ velocities are represented with *v*, broken lines) and the collisional c.m. coordinate (the reactants’ velocities are represented with *u*, solid lines). The ionic signals in the left and right sides respective to c.m. are defined as the forward and backward scatterings.

The velocity maps of C^+^ and O_2_^+^ products in Fig. 3 correspond to the ionic three-dimensional Newton spheres projected on the detector or reaction plane, rather than the central slices of the spheres. Nevertheless, we still can find the collision-energy dependences of the velocity distributions. Figure 3a indicates that the C^+^ yields are primarily located around the target CO_2_ at the lower collision energies, but most of them are distributed in the forward direction (namely the CO_2_ flying direction) at *E*_c.m._ = 4.75 eV. In contrast to the C^+^ velocity maps, as shown in Fig. 3b, most of the O_2_^+^ ions are always distributed around CO_2_ and become crowded at *E*_c.m._ = 4.75 eV. The forward scattering preference and the product distributions around the reactant CO_2_ indicate that the reactions would like to proceed in the large-parameter collisions, namely, the collisional or translational energy is hardly transferred to the target in such a contactless reaction. Therefore, the higher-threshold channel leading to the fragments 2 O(^3^P_2_) + C^+^(^2^P_1/2_) is unfavorable at *E*_c.m._ = 4.75 eV, due to the inefficient translational-to-internal energy transformation. The isotropic angular distributions (red circles in Fig. 3) represent the C^+^ yield of the CO_2_^+^ dissociation where CO_2_^+^ is assumed to be rebounded toward the c.m. position and receives 4.75 eV in a head-on collision. What we observed here obviously deviates from this hypothetical model.

The O_2_ coproduct is likely in *b*^1^Σ_g_^+^ at the lower collision energies, primarily due to near energy-resonance of the channel to O_2_(*b*^1^Σ_g_^+^) + C^+^(^2^P_1/2_) (see Fig. 1) and partially according to the velocity *u*(C^+^) distributions in Fig. S1a. The O_2_ coproduct could be in X̃^3^Σ_g_¯ or Ã^3^Σ_u_^+^ state at the higher collision energy 4.75 eV. The *u*(O_2_^+^) profiles in Fig. S1b, by contrast, exhibit a preference for the ground-state product O_2_^+^(X̃^2^Π_g_). O_2_ or O_2_^+^ in other states could be produced as the minor yields. More importantly, the absolute cross sections or production efficiencies of O_2_ and O_2_^+^ are the fundamental data of atmospheric reaction networks, but very few are available so far^18–20,24,26,28,29^. Extrapolating the total cross-sections of the He^+^ + CO_2_ reaction (all channels) from the higher collision energies^28,29^ to the low-energy range and according to the branching ratios obtained in this work, we further derive the absolute cross-sections of about 10^−22^ m^2^ of the reactions denoted with Eqs. (4) and (5) in a collision energy range of 1–5 eV (more details can be found in Supplementary Note 3). These cross-sections are a few hundred times larger than that of the direct production of O_2_ via DEA^7^.

**Fig. 4:**
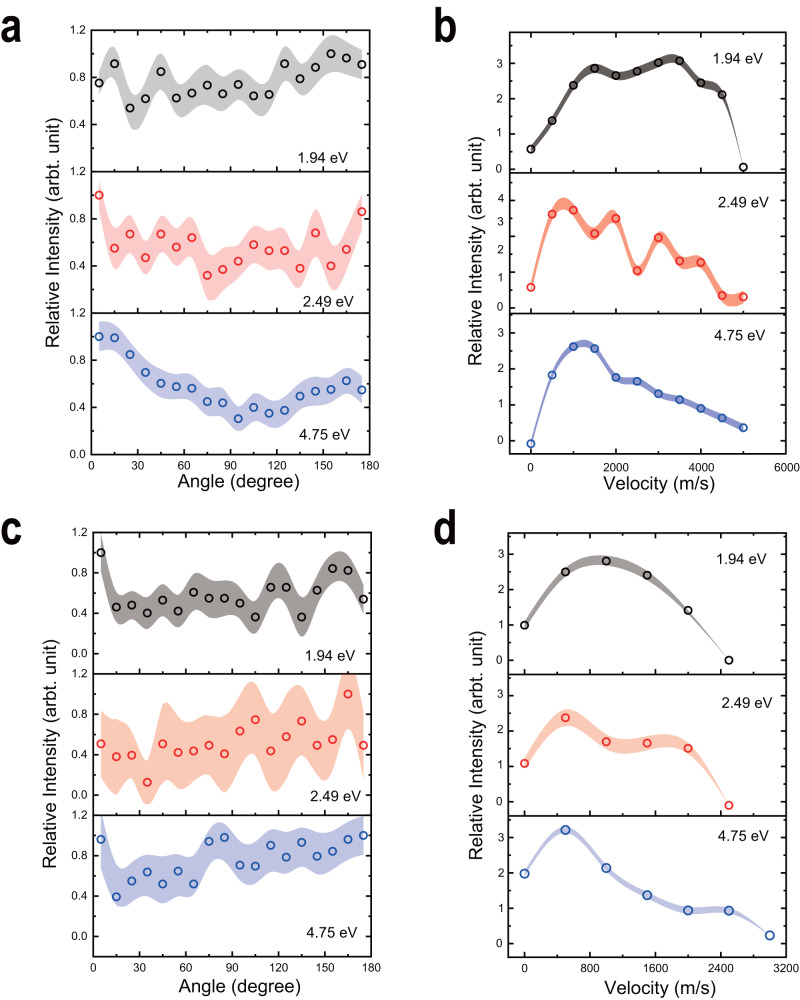
Angular and speed distributions of C^+^ (a, b) and O_2_^+^ (c, d) yields. These distributions after the independent intensity normalizations are plotted in the CO_2_ reaction coordinate by the transformation from those in the collisional c.m. coordinate and assuming the reactions lead to O_2_(*b*^1^Σ_g_^+^) + C^+^(^2^P_1/2_) (**a**, **b**) or O_2_^+^(X͂^2^Π_g_) + C(^3^P_0_) (**c**, **d**). The shades in different colors represent the statistical uncertainties and the ion intensities are normalized independently. Each point in the angular distribution (**a**, **c**) corresponds to all ion signals within a range of ±5°.

As discussed previously, oxygen-atom roaming and molecular bending are two feasible pathways to produce O_2_ from CO_2_, where the former usually results in the nearly isotropic angular distributions of the products while a distinct anisotropy can be observed for the latter^6,7^. As proposed above, in the large-parameter collisional reaction, the He atom formed by the prompt charge exchange should be a spectator in the subsequent dissociation. To further reveal this unimolecular-like process, we make a coordinate transformation from the reaction c.m. one (used in Fig. 3) to the CO_2_ reaction coordinate (more details can be found in Supplementary Note 4). As illustrated in Fig. S2, the CO_2_ reaction coordinate is also defined with respect to the collision axis, but its scattering angle is different from that in the reaction c.m. coordinate. Figure 4 shows the angular and velocity distributions of C^+^ and O_2_^+^ in the CO_2_ reaction coordinate.

Figure 4a exhibits the nearly isotropic distributions of C^+^ at the lower *E*_c.m._ values and a clearly forward preference at 4.75 eV, where O_2_ and C^+^ yields are assumed to be in specific states. As indicated in Fig. S3, this collision-energy dependence scarcely relies on the products’ states. A cartoon in the bottom panel of Fig. S3b illustrates a possible molecular orientation, namely, the dissociation undergoes the bended conformation which is almost perpendicular to the collision axis. It is reasonable that such a stereodynamics feature becomes indistinct in the slower collisional reaction. All O_2_^+^ angular distributions in Fig. 4c are almost isotropic, which could be attributed to the roaming dynamics, namely, the roaming O^+^ abstracts the oxygen atom from the CO moiety. The velocities of C^+^ and O_2_^+^ in Fig. 4b and d indicate the lower values at the higher collision energy, due to an enhancement of the products in the higher states.

To further understand the dissociation dynamics of CO_2_^+^, we plot the potential energy surfaces of C̃^2^Σ_g_^+^ state in Fig. S4 of Supplementary Note 5. Different shapes or patterns of above potential energy surfaces imply that the O_2_^+^ or O_2_ formation hardly undergoes a combinational motion of molecular bending and symmetric bond stretching, but possibly benefits from an asymmetric stretching. The asymmetric stretching should be also responsible for the predominance of the CO^+^ and O^+^ products observed in this work and the previous studies^18–20,24,26,28,29^. However, it would be a tough task to simulate the dynamic trajectories leading to O_2_ or O_2_^+^ because of their much lower yields. Furthermore, the nonadiabatic process, for instance, coupling between C̃^2^Σ_g_^+^ state and ^2^Π_u_-satellite states of CO_2_^+^^25^, may be involved in the reactions.

## Conclusions

Based on the previous and present findings, we make a conclusive remark on the origins of atmospheric O_2_. Since the production efficiency of the photodissociation of CO_2_ was not high enough to reproduce the measured data^30^, the other production sources of those O_2_ molecules must be considered. The He^+^ + CO_2_ reaction, particularly in the low collision energy range, is an important mechanism but was ignored before this study. Up to date, we have known different mechanisms for the origin of atmospheric O_2_. The O atoms produced in the ion-molecule reaction, DEA, and photodissociation can also participate in the formation of O_2_. Some contributions could be from the O^+^ and O_2_^+^ ions by the electron capture, however, these ions are likely responsible for the oxygen escaping^16,17^. All-inclusive networks of the atmospheric and interstellar oxygen-related reactions are expected to be established by more and more laboratory studies and astronomical observations.

## Methods

### Experiments

Our three-dimensional velocity map imaging apparatus has been described elsewhere^22^, in which the product ion detector consists of a set of multichannel plates and a Delay-Line-Detector (DLD *ϕ* = 80 mm, commercially available from Roentdek). The ion source that previously produced Ar^+^ ions^21^ was updated recently to produce lighter ions (such as He^+^). In the present experiment, the energy spreads (Δ*E*/*E* ~ 7%) of the He^+^ bunches are comparable to that of the Ar^+^ bunches. The *x−y* positions (the plane of the detector) and the time of flight (along *z* direction) of each ionic signal are recorded simultaneously with the DLD, and then a Newton sphere of a certain type of ionic yield is rebuilt with these data^22,23^. In Fig. 3, the two-dimensional images are plotted with the raw data of whole Newton spheres of the ionic products projected on the detector, where one pixel corresponds to ca. 200 m/s of C^+^ or O_2_^+^. On the other hand, the TOF mass spectrum can be directly obtained from the measurement of whole Newton spheres, thus the ion collection efficiency is nearly 100%. High-purity (99.99%) samples (He and CO_2_) are available commercially and used without further purification. The He^+^ reactant ions are produced in the electron impacts at 30 eV. The kinetic energies and the energy spreads of the reactants He^+^ and CO_2_ (its Δ*E*/*E* ~ 6%) were calibrated or determined prior to the experiments. During the experiments, the reaction chamber is evacuated under a steady vacuum condition of 2.0 × 10^−7^ mbar and the reactions happen in the field-free region. The working frequency is 10 kHz, and we need dozens of hours to record the velocity images at a given collision energy.

### Calculations

Equilibrium geometry and vibrational frequencies of the ground-state CO_2_ were calculated at the density functional theory level wb97xd/def2TZVP. This molecular geometry was used as the initial point in scanning the potential energy surfaces along the vibrational motions, i.e., symmetric stretching Q1, bending Q2, and asymmetric stretching Q3. Table S4 shows the corresponding values of real bond length and bond angle for each Q1–Q3. The equation of motion method based on the coupled cluster method limited to singles and doubles excitations and the basis set cc-pVTZ^31–34^ (encoded in Guassion16 suit of programs^35^) were used here to obtain the two-dimensional potential energy surfaces of CO_2_^+^(C^2^Σ_g_^+^).

### Supplementary information

Supplementary Information

## Data Availability

All data underlying the figures are deposited in the main text or as supplementary information. The calculated data about the potential energy surfaces of CO_2_^+^(C^2^Σ_g_^+^) are available upon the requests to the corresponding authors.
