# Supplementary material for: Direct production of molecular oxygen from carbon dioxide and helium ion collisions
Source: Commun Chem. 2023 Dec 6;6:267. doi: 10.1038/s42004-023-01074-2 (PMC10700575; doi:10.1038/s42004-023-01074-2)
Supplement: Supplementary file 1 — Supplementary Information [file 42004_2023_1074_MOESM1_ESM.pdf]

Supplementary Information of

Direct production of molecular oxygen from carbon dioxide and helium ion collisions

Yaya Zhi[a], Qiang Guo[a], Jingchen Xie[a], Jie Hu[b,]*, and Shan Xi Tian[a,b,c,]*

[a]*Department of Chemical Physics, Collaborative Innovation Center of Chemistry for Energy Materials (iChEM), University of Science and Technology of China, Hefei 230026, China*

[b]*Hefei National Research Center for Physical Sciences at the Microscale, University of Science and Technology of China, Hefei 230026, China*

[c]*Hefei National Laboratory, University of Science and Technology of China, Hefei 230088, China*

*Corresponding authors. Email: jiehu@ustc.edu.cn; sxtian@ustc.edu.cn

Supplementary Note 1. collisional kinetics

In the reactions,

$$He^{+} + CO_2 \rightarrow C^{+} + O_2 + He \qquad (1a)$$

$$He^{+} + CO_2 \rightarrow C + O_2^{+} + He \qquad (1b)$$

the atomic and molecular products can be in different electronic or rovibrational states. The energetic thresholds of above reactions together with the others[1] are listed in Table S1.

Table S1. Energetic thresholds (E_{th} in eV) with respect to the energies of the neutral reactants.

CO^{+} + O		O^{+} + CO		**C^{+} + O_2**		**C^{+} + 2O**		**O_2^{+} + C**	
States	E_{th}	States	E_{th}	States	E_{th}	States	E_{th}	States	E_{th}
$\tilde{X}^2\Sigma^+ + {}^3P_2$	19.4	${}^4S_{3/2} + \tilde{X}^1\Sigma^+$	19.1	${}^2P_{1/2} + \tilde{X}^3\Sigma_g^-$	22.8	${}^2P_{1/2} + {}^3P_2$	27.8	$\tilde{X}^2\Pi_g + {}^3P_0$	23.7
$\tilde{X}^2\Sigma^+ + {}^1D_2$	21.4	${}^2D_{5/2} + \tilde{X}^1\Sigma^+$	22.4	${}^2P_{1/2} + a^1\Delta_g$	23.7			$\tilde{X}^2\Pi_g + {}^1D_2$	24.9
$\tilde{A}^2\Pi + {}^3P_2$	22.0	${}^2P_{3/2} + \tilde{X}^1\Sigma^+$	24.1	${}^2P_{1/2} + b^1\Sigma_g^+$	24.4			$\tilde{X}^2\Pi_g + {}^1S_0$	26.4
$\tilde{X}^2\Sigma^+ + {}^1S_0$	23.6	${}^4S_{3/2} + a^3\Pi$	25.1	${}^2P_{1/2} + \tilde{A}^3\Sigma_u^+$	27.2			$a^4\Pi_u + {}^3P_0$	27.6
$\tilde{A}^2\Pi + {}^1D_2$	24.0	${}^4S_{3/2} + a^3\Sigma^+$	26.0	${}^4P_{1/2} + \tilde{X}^3\Sigma^-$	28.1				
$\tilde{B}^2\Sigma^+ + {}^3P_2$	25.1	${}^4S_{3/2} + \tilde{A}^1\Pi$	27.1						
$\tilde{X}^2\Sigma^+ + {}^5S_2$	28.6								

As shown in Figure 1, the O_2 yield could be in $\tilde{X}^3\Sigma_g^-$, $a^1\Delta_g$, or $b^1\Sigma_g^+$ state, irrespective of the energy transformation from the collisions. However, it is difficult to derive the state distribution of O_2 from the measurements of the co-product C^+. This difficulty arises from: three-body kinetics cannot be determined by the measurement of one product; possible populations of the rovibrational states of O_2 result in the quasi-continuous distributions of the C^+ kinetics; multiple pathways (if existing) also strongly influence the C^+ kinetics.

The following analyses are based on the assumptions: He atom is spectator in the dissociation of CO_2^+ after the prompt charge exchange; only the O_2 or O_2^+ are in the ro-vibrational ground state (but possibly in different electronic states). According to the energy conservation, we have

$$\mathrm{IP(He)} + E_{c.m.} = E_{int}(C^+) + E_{int}(O_2) + E_{int}(He) + E_k(C^+) + E_k(O_2) + E_k(He) \qquad (2a)$$

$$\mathrm{IP(He)} + E_{c.m.} = E_{int}(C) + E_{int}(O_2^+) + E_{int}(He) + E_k(C) + E_k(O_2^+) + E_k(He) \quad (2b)$$

where E_{int} represents the energy difference between the electronically excited (or ionization) state and the ground state of the neutral (cation), and E_k is the kinetic energy of the product in the center-of-mass coordinate of reaction. As the spectator, E_k(He) equals $E_k(He^+)$; E_{int}(He) = 0 eV since $He^*(^3S)$ cannot be produced in the present experiments. $E_{c.m.} = E_k(He^+) + E_k(CO_2)$.

If the electronically ground state O_2 ($X^3\Sigma_g^-$) is produced in channel 1a, we will have,

$$\mathrm{IP(He)} + E_k(CO_2) = E_{int}(C^+) + E_k(C^+) + E_k(O_2) \quad (3)$$

where $E_{int}(C^+)$ equals the ionization potential of atomic carbon. For the spectator He, the dissociation is satisfied with the momentum conservation,

$$\mathrm{m}(\mathrm{CO_2^+})\mathbf{u}(\mathrm{CO_2^+}) = \mathrm{m}(\mathrm{C^+})\mathbf{u}(\mathrm{C^+}) + \mathrm{m}(O_2)\mathbf{u}(O_2) \quad (4)$$

In combination of eqs. (3) and (4), we can obtain the highest velocity or kinetic energy of C^+. Accordingly, we can also obtain that of O_2^+ for channel 1b. The estimations for the products in the energetically permitted states are labelled in Figure S1. The C^+ or O_2^+ yields with the velocities lower than that of the target CO_2 (vertical green line) are attributed to those flying backward.

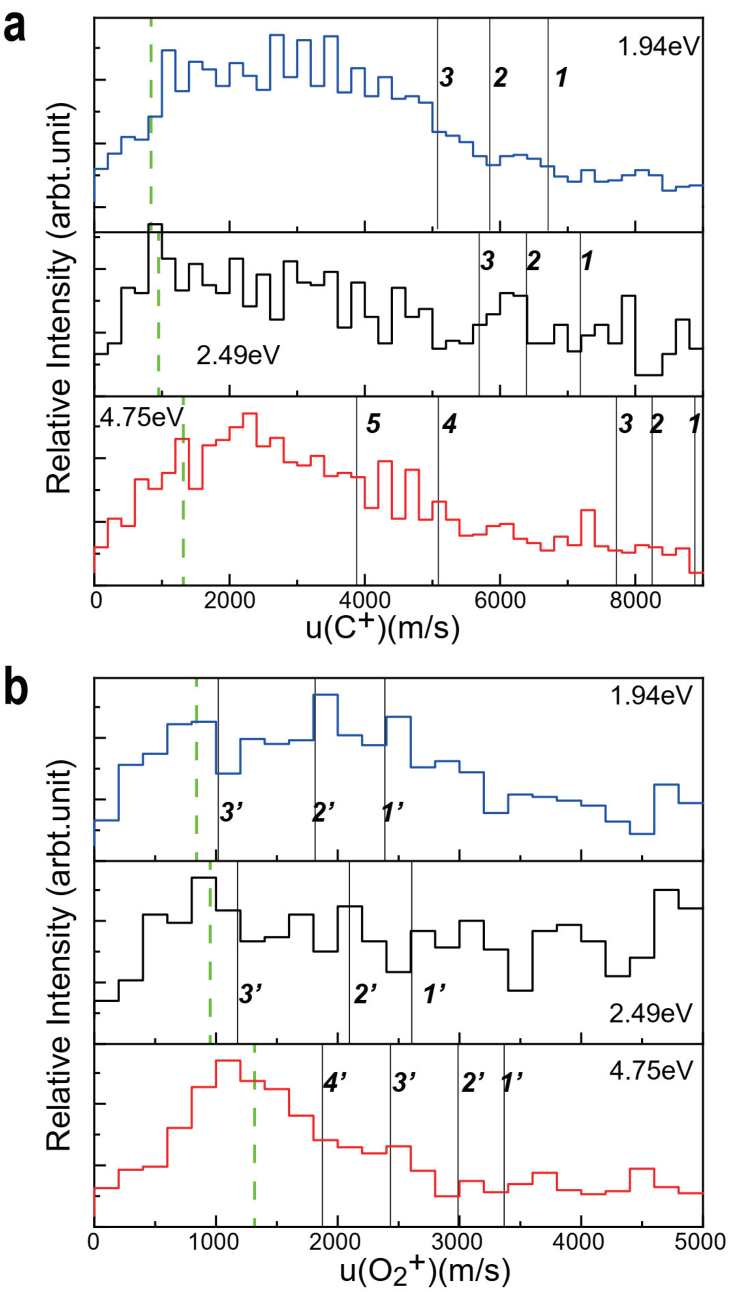

Figure S1. Velocity distributions of the C^+(a) and O_2^+ (b) yields. The velocities of CO_2 are denoted with green broken lines. **a**, The maximum velocities of C^+ are labelled with vertical lines for the reactions leading to $O_2(\tilde{X}^3\Sigma_g^-)$ + $C^+(^2P_{1/2})$ (***1***), $O_2(a^1\Delta_g)$ + $C^+(^2P_{1/2})$ (***2***), $O_2(b^1\Sigma_g^+)$ + $C^+(^2P_{1/2})$ (***3***), $O_2(\tilde{A}^3\Sigma_u^+)$ + $C^+(^2P_{1/2})$ (***4***), and $O_2(\tilde{X}^3\Sigma_g^-)$ + $C^+(^4P_{1/2})$ (***5***). **b**, The maximum velocities of O_2^+ are labelled with vertical lines for the reactions leading to $O_2^+(\tilde{X}^2\Pi_g)$ + $C(^3P_0)$ (***1'***), $O_2^+(\tilde{X}^2\Pi_g)$ + $C(^1D_2)$ (***2'***), $O_2^+(\tilde{X}^2\Pi_g)$ + $C(^1S_0)$ (***3'***), and $O_2^+(a^4\Pi_u)$ + $C(^3P_0)$ (***4'***). The ion intensity in each profile is normalized independently.

Supplementary Note 2. branching ratios

There are different channels in the reactions of He^+ with CO_2, even at a thermal collisional energy[2-4]. Since the dissociative charge exchange channels (to produce CO^+ + O, O^+ + CO, O_2^+ + C, C^+ + O_2) are exothermic[1], the pure charge exchange is rare although it is also exothermic. In Table S2, we make a comparison of the branching ratios between the present results and the previous measurements[2-4]. It is noted that the branching ratios for CO_2^+ could be overestimated due to the secondary collisions in the previous measurements[2-4]. In the present crossed-beam experiment, there is no such problem. From the TOF mass spectra, the peak areas of different ionic yields are obtained, then the branching ratios of the different channels are estimated and listed in Table S2.

Table S2. Branching Ratios of the Collisional Reactions of He^+ + CO_2

Ionic Yield	Present work (Different Collision Energies)			Literatures (Thermal Energy)		
	4.75 eV	2.49 eV	1.94 eV	Ref. 2	Ref. 3	Ref. 4
CO_2^+	1.8±0.2%	1.4±0.2%	1.1±0.2%	≤1%	11%	5%
O_2^+	0.2±0.2%	0.5±0.2%	0.7±0.2%	≤1%	1%	-
CO^+	83.9±0.2%	83.0±0.2%	82.0±0.3%	77%, 82%	79%	80%
O^+	13.9±0.2%	14.7±0.2%	15.4±0.2%	18%, 16%	9%	15%
C^+	0.2±0.2%	0.4±0.2%	0.8±0.2%	4%, 1%	-	-

Supplementary Note 3. absolute cross sections

Absolute cross section and reaction rate are the important data to simulate complicated physicochemical networks. The cross sections of the reactions leading to O_2 (corresponding to C^+) and O_2^+ should be useful to establish a general circulation model of atmospheric oxygen-related reactions. Up to date, absolute cross sections of He^+ + CO_2 reaction in the present collision energy range are unavailable. Extrapolating the total cross sections of He^+ + CO_2 reaction (including pure charge exchange and dissociative charge exchange processes) from the higher (few keV) or much lower (from hundreds to thousands of km/s of He^+ velocity in the c.m. frame) collision energies[5,6], we derive the absolute cross sections in the collision energy of 1-5 eV (about ten km/s or less of He^+ velocity), namely, the upper and lower limits of cross sections. These values can be found in Table S3, and the cross sections of the dissociative charge exchange reactions leading to O_2 or O_2^+ product around 10^{-22} m^2 are proposed. These cross sections are a few hundred times higher than the O_2 production of dissociative electron attachment[7]. The absolute cross section or reaction rate of the photodissociation $hv + CO_2 \rightarrow C + O_2$ is unavailable[8]. The real contributions of above reactions to the atmospheric origin of O_2 are also dependent on the quantity of He^+, electron, and photon at the specific energies. To the best our knowledge, a large quantity of low-energy He^+ and electron have been detected in the upper atmospheres[9,10].

Table S3. Estimated values (upper-lower limits) of the cross sections of He^+ + CO_2 reactions.

Collision Energy	C^+ yield (m^2)	O_2^+ yield (m^2)	Total (m^2)
5.00 eV	–	–	30.15-3.25 x10^{-20}
4.75 eV	7.5-0.8 x 10^{-22}	7.5-0.8 x 10^{-22}	30.18-3.24 x10^{-20}
2.49 eV	1.2-0.1 x 10^{-21}	1.5-0.2 x 10^{-21}	30.30-3.17 x10^{-20}
1.94 eV	2.5-0.3 x 10^{-21}	2.5-0.2 x 10^{-21}	30.33-3.14 x10^{-20}
1.00 eV	–	–	30.37-3.05 x 10^{-20}

Supplementary Note 4. coordinate transformation

The collision or scattering dynamics of ion-molecule reaction are usually diagrammatized and analyzed in the collisional coordinate of center-of-mass of two reactants, as depicted in Figure 3. Here and in many literatures, the flying direction of the neutral molecule (target) is defined as 'forward' and the opposite (or the flying direction of the ionic reactant) is the direction of 'backward'. If the He is spectator during the CO_2^+ dissociation [i.e., $\boldsymbol{u}(CO_2^+) = \boldsymbol{u}(CO_2)$], we redefine the forward and backward directions in the coordinate of CO_2 or CO_2^+. As shown in Figure S2, the forward scattered yields should have the θ' values (in the CO_2 reaction coordinate) less than 90°.

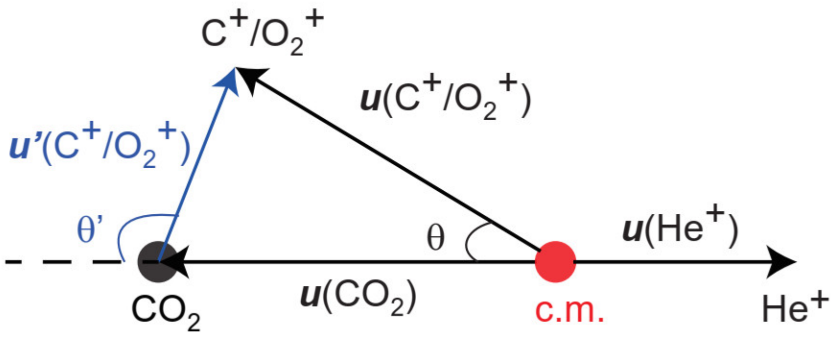

Figure S2. Coordinate transformation. The velocity of the ionic product $\boldsymbol{u}(C^+/O_2^+)$ in the reaction coordinate of center-of-mass (c.m.) is represented with $\boldsymbol{u}'(C^+/O_2^+)$ in the coordinate of CO_2 or CO_2^+ by the coordinate transformation. θ and θ' are the scattering angles in the reaction and CO_2 coordinates, respectively.

By the coordinate transformation described in Figure S2, the velocity images of Figure 3 are redrawn in the CO_2 coordinate. Then, the angular (θ') distributions of C^+ and O_2^+ are plotted in Figure 4. Here, we did not select the ions in some specific velocity range, while the ions with all velocities were included to plot the angular (θ') distributions. Due to the energy conservation, the C^+ velocity-image size for the channel leading to $O_2(a^1\Delta_g) + C^+(^2P_{1/2})$ is smaller than that for the channel leading to $O_2(\tilde{X}^3\Sigma_g^-) + C^+(^2P_{1/2})$. However, such a size change scarcely influences the angular (θ') distribution. By comparing the angular distributions in Figures 3a, S3a, and S3b, one can find that the features are nearly unchanged or independent on the products' states. On the other hand, all of these angular

distributions show the forward distribution (θ' < 30°) becomes more favorable in the reaction of 4.75 eV.

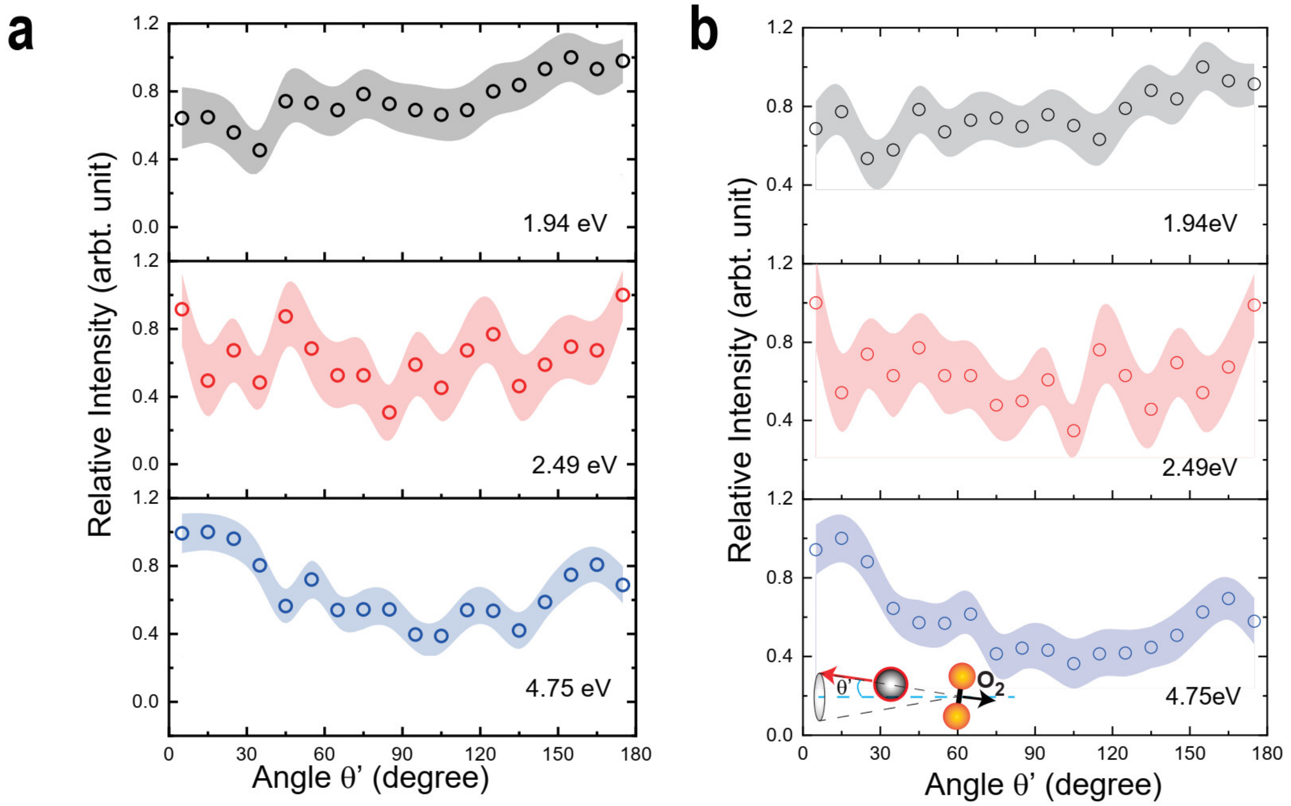

Figure S3. Angle distributions of the C^+ yields in the coordinate of CO_2. a) Assuming that the dissociation leads to $O_2(\tilde{X}^3\Sigma_g^-)$ + $C^+(^2P_{1/2})$. **b**) Assuming that the dissociation leads to $O_2(a^1\Delta_g)$ + $C^+(^2P_{1/2})$. At the higher collision energy (4.75 eV), the C^+ yields are preferably scattered forward.

Supplementary Note 5. potential energy surfaces

Table S4 shows the corresponding values of real bond length and bond angle for symmetric stretching Q1, bending Q2, and asymmetric stretching Q3 of CO_2. The two-dimensional PESs of $CO_2^+(C^2\Sigma_g^+)$ are plotted in Figure S4. Noted that the method used here, i.e., the equation of motion method based on the coupled cluster method limited to singles and doubles excitations, has been evaluated[11] and the average absolute errors in the predictions of molecular electronically excited states were 0.05eV-0.1eV.[11,12] In this work, we do not aim to obtain the PESs with the high numerical accuracy, but pay attention to the pattern or profile differences between Figures S4a and S4b.

Table S4. Corresponding values of the bond length or bond angle of Q1, Q2, and Q3.

normal coordinates	0	1	2	3	4	5	6	7	8	9	10
Q1/bond length Å	1.157	1.228	1.299	1.370	1.441	1.512	1.583	1.654	1.725	1.796	1.867
Q2/bond angle Degree	180.0	168.7	157.7	147.0	136.9	127.4	118.7	110.7	103.4	96.8	90.7
Q3/bond length Å	1.157<br>1.157	1.036<br>1.306	0.915<br>1.315	0.794<br>1.323	0.673<br>1.332	0.552<br>1.340	-	-	-	-	-

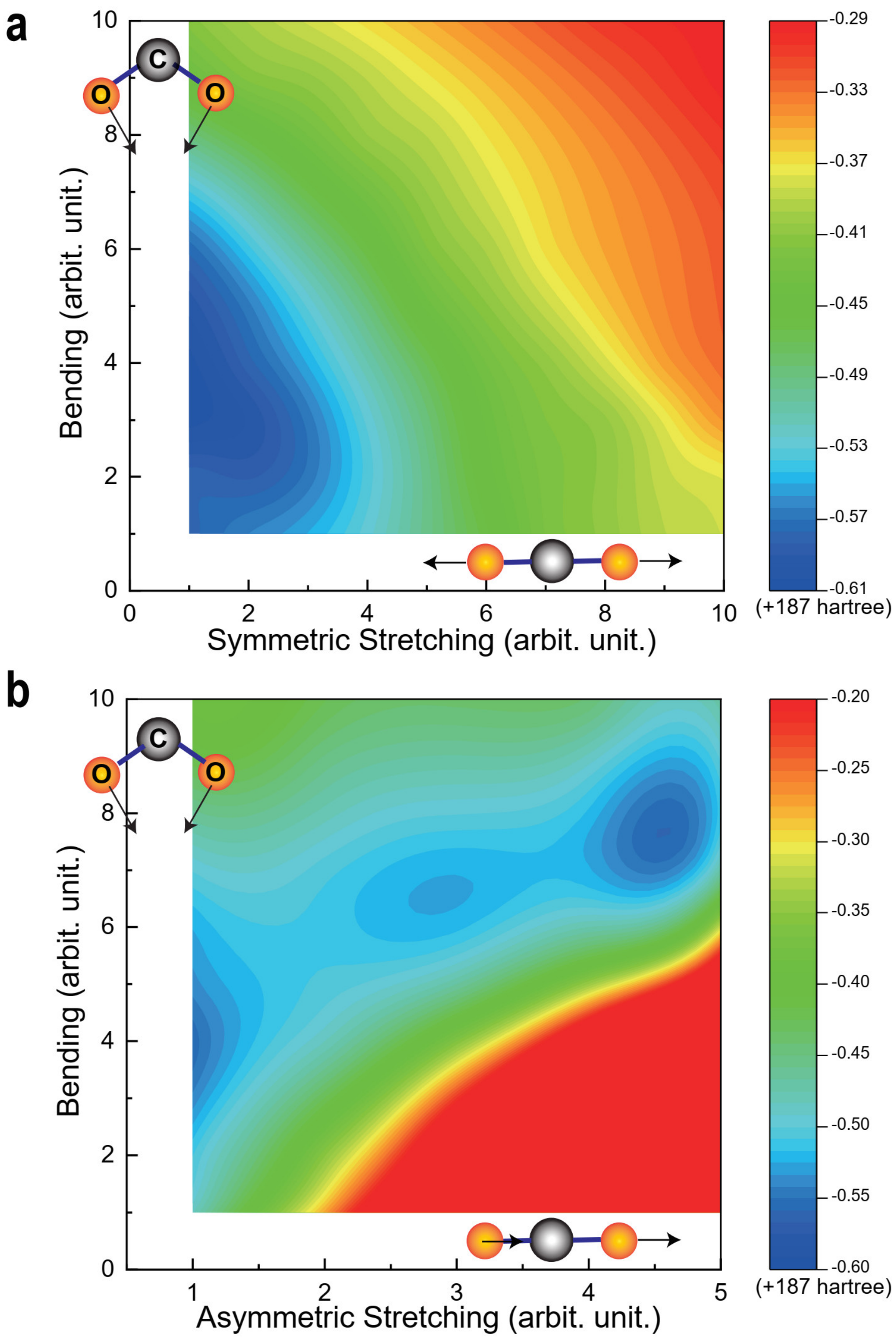

Figure S4. Potential energy surfaces of $CO_2^+(C^2\Sigma_g^+)$. The surfaces predicted with EMO-CCSD method are plotted in terms of two fundamental vibrational motions. **a**, Symmetric stretching v.s. Bending. **b**, Asymmetric stretching v.s. Bending.

Supplementary References

1. McMillan, M. R. & Coplan, M. A. Kinematics of He^+-CO_2 dissociative charge exchange. *J. Chem. Phys.* **71**, 3063- 3071 (1979).

2. Anicich, V. G., Laudenslager, J. B., Huntress Jr., W. T. & Futrell, J. H. Product distributions for some thermal energy charge transfer reactions of rare gas ions. *J. Chem. Phys.* **67**, 4340- 4350(1977).

3. Adams, N. G. & Smith, D. Product-ion distributions for some ion-molecule reaction. *J. Phys. B: At. Mol. Phys.* **9**, 1439-1450 (1976).

4. Fehsenfeld, F. C., Schmeltekopf, A. L., Dunkin, A. L. & Ferguson. E. E. ESSA Technical Report ERL **135**, 5 (1969).

5. Parker, J. E. & Johnson, C. A. F. A two-state treatment of the electron transfer reactions from carbon dioxide to helium ions. *Int. J. Mass Spectrom. Ion Proc.* **94**, 87-99 (1989).

6. Werbowy, S. & Pranszke, B. Charge-exchange processes in collisions of H^+, H_2^+, H_3^+, He^+, and He_2^+ ions with CO and CO_2 molecules at energies below 1000 eV. *Phys. Rev. A*. **93**, 022713 (2016).

7. Wang, X.-D., Gao, X.-F., Xuan, C.-J. & Tian, S. X. Dissociative electron attachment to CO_2 produces molecular oxygen. *Nat. Chem.* **8**, 258-263 (2016).

8. Lu, Z., Chang, Y.-C., Yin, Q.-Z., Ng, C. Y. & Jackson, W. M. Evidence for direct molecular oxygen production in CO_2 photodissociation. *Science* **346**, 61-64 (2014).

9. Yoshikawa, I. et al. Development of a compact EUV photometer for imaging the planetary magnetosphere, *J. Geophys. Res.* **106**, 26057 (2001).

10. Larsson, M., Geppert, W. D. & Nyman, G. Ion chemistry in space, *Rep. Prog. Phys.* **75**, 066901 (2012).

11. V., Perera, A., Nooijen, M. & Bartlett, R. J. Excited states from modified coupled cluster methods: Are they any better than EOM CCSD? *J. Chem. Phys.* **146**, 144104 (2017)

12. Goings, J. J., Caricato, M., Frisch, M. J. & Li, X. Assessment of low-scaling approximations to the equation of motion coupled-cluster singles and doubles equations. *J. Chem. Phys.* **141**, 164116 (2014).
